# A 15‐year population‐based study on incidence and vaccination coverage in pediatric inflammatory bowel disease in Italy

**DOI:** 10.1002/ped4.70023

**Published:** 2025-09-18

**Authors:** Francesca Fortunato, Angelo Campanozzi, Michele Di Toma, Alessandra Marinari, Domenico Martinelli

**Affiliations:** ^1^ Department of Medical and Surgical Sciences Hygiene Unit University of Foggia Foggia Italy; ^2^ Department of Medical and Surgical Sciences Pediatrics Unit University of Foggia Foggia Italy; ^3^ Department of Pediatrics Policlinico Foggia Hospital Foggia Italy

**Keywords:** Crohn's disease, Incidence, Inflammatory bowel disease, Pediatrics, Ulcerative colitis, Vaccination coverage

## Abstract

**Importance:**

The incidence of pediatric inflammatory bowel disease (IBD) is increasing worldwide, particularly after the coronavirus disease 2019 pandemic. As children with IBD are at higher risk of infection, adherence to vaccination schedules is essential. However, data on the incidence of IBD and vaccination coverage in Italian children remain limited.

**Objective:**

To assess the incidence of IBD in children aged 0–14 years in the Apulia region of Italy (2009–2023) and to evaluate their vaccination coverage for both mandatory and recommended vaccines.

**Methods:**

A retrospective population‐based study was conducted using hospital discharge records and a user fee exemption registry to identify incident IBD cases. Vaccination history was obtained from the regional immunization system. Age‐standardized incidence rates (SIRs) were calculated. Vaccination coverage was assessed based on the receipt of all age‐appropriate vaccines according to regional immunization schedules.

**Results:**

Between 2009 and 2023, 1044 incident pediatric IBD cases were identified, with an average annual SIR of 12.7/100 000. A significant increase in incidence occurred after 2020 (*P* < 0.001), peaking in the 10–14 age group. Among the 259 patients assessed for vaccination coverage, the rates were high for hexavalent (100.0%) and pneumococcal vaccines (99.2%), moderate for rotavirus (82.5%) and meningococcal vaccines (79.9%), and lower for human papillomavirus (66.3% in females and 52.1% in males) and diphtheria‐tetanus‐acellular pertussis and inactivated polio vaccines (dTap‐IPV) booster (30.2%). Influenza coverage in 2023/24 was 18.9%. Children with complex chronic conditions had lower uptake of measles, mumps, and rubella and dTap‐IPV boosters.

**Interpretation:**

The incidence of pediatric IBD is increasing in Apulia, whereas vaccination coverage remains below optimal levels. Targeted strategies are needed to enhance immunization in this vulnerable population.

## Graphical abstract

Increasing incidence of pediatric inflammatory bowel disease (IBD) and suboptimal vaccination coverage in Apulia, Italy (2009–2023). A total of 1044 incident cases of paediatric IBD were identified in the period 2009–2023 in Apulia, Italy, giving an average annual standardized incidence rate of 12.7/100 000. A significant increase in incidence occurred after 2020 (incidence rate ratio = 3.7, *P* < 0.001), peaking in the 10–14 age group. Of the 259 patients assessed for vaccination coverage, uptake was high for the hexavalent and the pneumococcal vaccines, moderate for the rotavirus and meningococcal vaccines, and lower for the human papillomavirus vaccine and diphtheria‐tetanus‐acellular pertussis and inactivated polio vaccines booster. Influenza coverage in 2023/24 was the lowest. Targeted strategies are needed to improve immunisation rates in this vulnerable population.

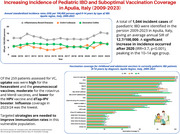

## INTRODUCTION

Crohn's disease (CD) and ulcerative colitis (UC) are chronic inflammatory bowel diseases (IBD) that usually present during adolescence or early adulthood.^1^, ^2^ Nearly 25% of IBD cases are diagnosed before the age of 20 years, with a significant proportion present in childhood. Around 4% of pediatric patients are diagnosed before the age of 5, and 18% before the age of 10, with incidence peaking during adolescence.^2^ Several recent studies reported an increasing global burden of pediatric IBD, with a steady rise in incidence over the past two decades.^3^, ^4^, ^5^, ^6^, ^7^ In 2019, the number of new IBD cases in children and adolescents worldwide reached 25 659, an increase of 22.8% and 18.5% respectively compared to 1990.^7^, ^8^ Even in Italy, new diagnoses of IBD rose from 175 to 219 per year between 2009 and 2018, with UC being the most common form.^9^ Moreover, recent studies have reported an increase in the incidence of IBD in children worldwide following the coronavirus disease 2019 (COVID‐19) pandemic.^4^, ^10^, ^11^ Despite the increasing incidence of IBD, there is a significant lack of robust epidemiological data for pediatric and adolescent populations.^1^ In the case of Italy, current incidence estimates are mostly derived from geographically restricted cohorts, which limits the comprehensive epidemiological characterization of these populations.^12^

The increasing incidence of pediatric IBD highlights the critical importance of vaccination in reducing the risk of infection in immunocompromised patients receiving immunosuppressive therapies such as corticosteroids, immunomodulators, or tumor necrosis factor‐alpha inhibitors.^13^, ^14^, ^15^, ^16^ Although these therapies are effective in controlling inflammation, they increase susceptibility to vaccine‐preventable diseases (VPDs), which continue to cause significant morbidity in this population. The most common pathogens associated with serious outcomes are varicella and influenza viruses, human papillomavirus (HPV), and *S. pneumoniae*.^15^, ^17^, ^18^, ^19^, ^20^, ^21^ Current international guidelines recommend that all children with IBD receive a vaccination schedule similar to that of the general population.^14^, ^15^, ^22^, ^23^ Nevertheless, vaccination coverage rates in IBD patients consistently fall below population‐based targets and often show lower adherence than immunocompetent populations.^13^, ^14^ Furthermore, the lack of studies specifically evaluating vaccination coverage in pediatric IBD cohorts limits a comprehensive understanding of vaccination adherence patterns and associated infection risk profiles in this immunocompromised population subgroup.

The primary objective of this study was to evaluate the incidence of IBD in the pediatric population in the Apulia Region of Italy between 2009 and 2023. In addition, this study aimed to assess the vaccination coverage rates of key vaccines, both mandatory and recommended by the Italian Vaccine Prevention Plan, among pediatric patients with IBD.

## METHODS

### Ethical approval

This study was conducted in accordance with the Declaration of Helsinki (1975, revised 2008). The study protocol was approved by the Local Ethics Committee (“Interprovinciale Area‐I‐AOU‐Foggia‐ASL‐FG‐ASL‐BAT”) (ref.10/CE/2025). According to the recent reform of Article 110 of the Privacy Code (Article 44, paragraph 1‐bis of law no.56 of April 29, 2024, converting the decree law no.19 of March 2, 2024) concerning the processing of personal data for medical, biomedical, and epidemiological research purposes relating to deceased or untraceable patients (Register of Measures No. 298 of May 9, 2024), informed consent was not explicitly required from the subjects enrolled in the study, as it was designed as a large retrospective cohort design and it was not feasible to contact all eligible individuals. Nevertheless, a detailed Data Protection Impact Assessment was conducted according to Article 35 of Reg.UE 2016/679, where all data were processed and analyzed in an anonymized form to ensure that individuals could not be identified.

### Study design, setting, and population

This retrospective population‐based study of children aged 0–14 years with a confirmed diagnosis of IBD between 2009 and 2023 was conducted in the Puglia region of Italy.

In line with international and national guidelines,^14^, ^22^, ^24^, ^25^, ^26^ the Puglia Regional Immunization Prevention Plans^27^, ^28^, ^29^, ^30^, ^31^, ^32^, ^33^ require that patients with IBD receive a complete immunization schedule identical to that recommended for the general pediatric population. The plans prioritize the administration of diphtheria, tetanus, and acellular pertussis (DTaP/dTap) vaccines, inactivated polio vaccines (IPVs), *Haemophilus influenzae* type b (Hib) vaccines, pneumococcal conjugate vaccines (PCVs), meningococcal vaccines (quadrivalent ACWY, MenB, and MenC), hepatitis viruses (A and B) (HAV and HBV), measles, mumps, and rubella (MMR), varicella (V), and HPV vaccination series. Additionally, annual influenza vaccination and age‐appropriate dTap booster doses are classified as high‐priority interventions for immunologically vulnerable population.^27^, ^28^, ^29^, ^30^, ^31^, ^32^, ^33^

### Data sources

Incident IBD cases were identified from two primary data sources: the Hospital Discharge Registry (HDR) and the User Fee Exemption Registry (UFER). The vaccination history of IBD patients was retrieved from the Apulian Immunization Information System (IIS).

The HDR collects data on discharge diagnoses (one main and up to five secondary diagnoses) and procedures for all hospitalized patients, coded using the International Classification of Diseases, Ninth Revision, Clinical Modification (ICD‐9‐CM). The UFER contains information on all persons with chronic conditions/illnesses who are entitled to free medical consultations and medicines in a given period; each chronic condition is identified by a unique code and a unique date of diagnosis. The IIS is a confidential, population‐based, computerized database that records all immunization doses administered by vaccination providers to residents of the Apulia region. In all these sources, the subjects were identified by a personal ID number.

### Participants

**FIGURE 1:**
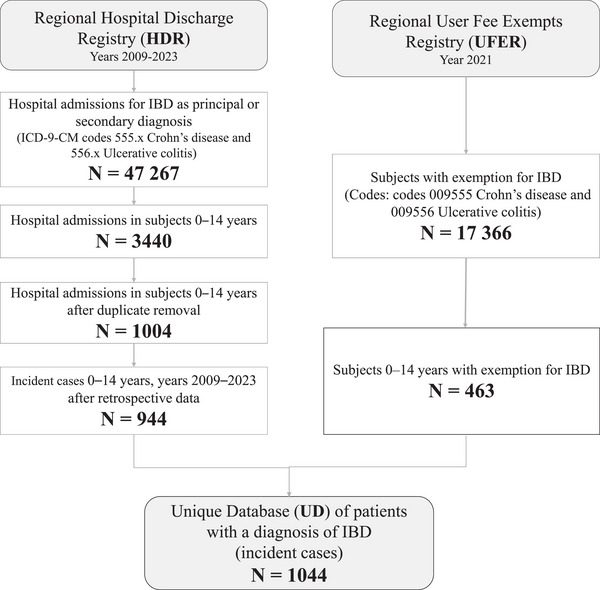
Flowchart for the identification of incident IBD cases in the Hospital Discharge Registry (HDR) and the User Fee Exemption Registry (UFER), Apulia region, Italy, 2009–2023. IBD, inflammatory bowel disease.

Incident IBD cases were defined as Apulian children aged 0–14 years diagnosed with CD and UC between January 1, 2009, and December 31, 2023, and registered in the HDR and/or UFER. In the HDR (databases 2009–2023), cases were retrieved by searching the ICD‐9‐CM codes 555.x CD and 556.x UC in either a principal or secondary diagnosis field. If a case was reported more than once in the HDR with different dates of admission, only the entry with the earliest date was included, and these data were used as a proxy for the date of diagnosis. Moreover, to ensure that only new IBD diagnoses were extracted, data cleaning was performed by comparing data from the period 2009–2023 with that from 2001 to 2008, identifying duplicates by using the personal ID number as the linkage key. The quality of matching was assessed by estimating the linkage error rates to understand the mechanisms by which these errors may have affected and biased the results. In UFER (dataset 2021), the search codes for cases with diagnosis data from January 1, 2009, to December 31, 2023, were 009.555 CD and 009.556 UC. Records of patients with missing personal ID numbers were excluded. A Unique Database (UD) was created by matching the records extracted from the two data sources by using the personal ID number as the linkage key (Figure 1). If a case was recorded in both the HDR and UFER, the case was recorded once in the UD with the earlier date of diagnosis.

Subsequently, the UD database 2009–2023 was linked with the Apulian HDR from 2001 to 2023 to collect information on chronic comorbid conditions and history of prematurity. Chronic conditions were classified using the pediatric Complex Chronic Conditions (CCCs) classification system (v2),^34^ that represents the “gold standard” for classifying children with comorbidities. Such a method uses the diagnosis codes of the ICD‐9‐CM or ICD‐10‐CM to create CCC categories that have a high likelihood of meeting the CCC definition: neuro‐muscular, cardiovascular, congenital, respiratory, gastrointestinal, renal, metabolic, haematological, cancer, and a category of perinatal conditions (premature and neonatal).^34^ Children with any of these nine conditions are considered to have a CCC.^34^

Finally, incident IBD cases aged 0–14 years on December 31, 2024, from UD were linked with their individual immunization records in IIS, using the personal ID number as the identifier. IBD cases were classified as fully vaccinated if they received all age‐specific doses recommended for each birth cohort in the Regional Immunization Plans as they evolved since 2009 (Table S1).^27^, ^28^, ^29^, ^30^, ^31^, ^32^, ^33^ Cases were excluded from the analysis if the vaccination status could not be retrieved from the IIS.

### Variables and measurement

Age‐standardized incidence rates (SIRs) stratified by sex, age group (0–4, 5–9, and 10–14 years of age at diagnosis), year of diagnosis, and type of IBD (CD and UC) were calculated by dividing the number of cases from UD by the number of inhabitants in the Apulia Region per 100 000 in the period 2009–2023. Direct standardization was performed using the European population of 2013 as a reference.^35^ The mid‐year estimates of the Apulian population were obtained from the ISTAT (Italy's National Census Bureau) estimate.

Vaccination coverage was calculated as the number of vaccinated cases divided by the total number of IBD cases. More specifically, according to the evolving regional immunization plan, vaccination coverage was calculated for DTaP‐IPV‐HBV‐Hib, PCV, MenB, and rotavirus primary schedules as recommended among infants in the first year of life, one dose of MenC/MenACWY, and two doses of HAV in toddlers in the second year of life; DTaP‐IPV/dTap‐IPV first booster and two doses of MMRV or MMR+V in children aged 5/6 years; HPV full schedule and dTap‐IPV second booster in adolescents up to 14 years of age (Table S1). For influenza, vaccination coverage was calculated from the 2020/2021 season, when the Puglia region began recommending vaccination for all children older than six months. Additionally, we calculated the vaccination coverage for the full two‐dose cycle of the COVID‐19 vaccine in the eligible IBD population (aged ≥5 years) from 2021 to 2023.

### Statistical analysis

Descriptive statistics were computed. Categorical variables (sex, age group, year of diagnosis, and type of IBD) were expressed as counts and percentages in each category. Continuous variables (i.e., age) are expressed as medians (interquartile range [IQR]). Ninety‐five confidence intervals (95% CIs) around the SIRs were calculated, assuming a Poisson distribution.

To assess the difference in incidence rates between males and females, among age groups, and between the pre‐COVID‐19 period (2009–2019) and the post‐COVID‐19 period (2020–2023), Poisson regression models were performed, and incidence rate ratios (IRRs) were calculated with 95% CI, with *P*‐values < 0.05 considered significant.

Univariate analysis was performed to assess the association between vaccination status and CCC, using double‐entry contingency tables and calculating chi‐squared and odds ratios (ORs) with 95% CI, with *P*‐values < 0.05. Variables significantly associated with vaccination were included in a multivariate logistic model to calculate adjusted ORs with 95% CI. Data were analyzed using Stata MP 18.0 for Mac OS software.

## RESULTS

Between January 1, 2009, and December 31, 2023, a total of 47 267 hospitalizations for IBD occurred in the Apulia region (CD: 21 712; UC: 25 555), of which 3440 (CD: 1700; UC: 1740) were aged 0–14 years. In 2021, 17 366 individuals were recorded as eligible for fee exemption due to IBD (CD: 5386; UC: 11 980) in UFER, including 463 patients (CD: 162; UC: 301) 0–14 years of age with diagnostic data from January 1, 2009, to December 31, 2023 (Figure 1).

After data processing and duplicate removal, 1044 cases (approximately 70 per year) were included in the study for incidence estimates (Figure 1). Of these, 54.0% (*n* = 564) were male, with a median age of 11 years. More than 60% (*n* = 642) were diagnosed between the ages of 10 and 14 years. At least one chronic comorbidity condition was documented in 272 (26.1%) patients (Table 1).

**TABLE 1:** Demographics and complex chronic conditions (CCC) of pediatric inflammatory bowel disease (IBD) cases aged 0–14 years, Apulia region, Italy, 2009–2023

Variables	CD (*n* = 460)	UC (*n* = 584)
Sex		
M	267 (58.0)	297 (50.9)
F	193 (42.0)	287 (49.1)
Age at IBD diagnosis (years)	11 (8–13)	11 (7–13)
Age group at IBD diagnosis (years)		
0–4	61 (13.3)	81 (13.9)
5–9	113 (24.5)	147 (25.2)
10–14	286 (62.2)	356 (60.9)
Year at IBD diagnosis		
2009	37 (8.0)	36 (6.2)
2010	30 (6.5)	42 (7.2)
2011	43 (9.3)	47 (8.0)
2012	31 (6.7)	48 (8.2)
2013	28 (6.1)	45 (7.7)
2014	19 (4.1)	43 (7.4)
2015	20 (4.3)	43 (7.4)
2016	25 (5.4)	41 (7.0)
2017	29 (6.3)	32 (5.5)
2018	31 (6.7)	29 (5.0)
2019	28 (6.1)	30 (5.1)
2020	35 (7.6)	24 (4.1)
2021	26 (5.7)	44 (7.5)
2022	38 (8.3)	43 (7.4)
2023	40 (8.7)	37 (6.3)
Complex chronic conditions (CCCs)^a^	127 (27.6)	145 (24.8)
Categories of CCCs		
Neurologic and neuromuscular	7 (1.5)	11 (1.9)
Cardiovascular	10 (2.2)	10 (1.7)
Respiratory	22 (4.8)	19 (3.3)
Renal and urologic	8 (1.7)	12 (2.1)
Gastrointestinal (excluding CD and UC)	26 (5.7)	38 (6.5)
Hematologic or immunologic	39 (8.5)	26 (4.5)
Metabolic	14 (3.0)	21 (3.6)
Other congenital or genetic defect	12 (2.6)	15 (2.6)
Malignancy	7 (1.5)	7 (1.2)
Premature and neonatal	21 (4.6)	24 (4.1)
Miscellaneous, not elsewhere classified	0	2 (0.3)

Data were shown as *n* (%) or median (IQR).

^a^ At least one comorbidity. Abbreviations: IBD, inflammatory bowel disease; CD, Crohn's disease; UC, ulcerative colitis; M, male; F, female.

**FIGURE 2:**
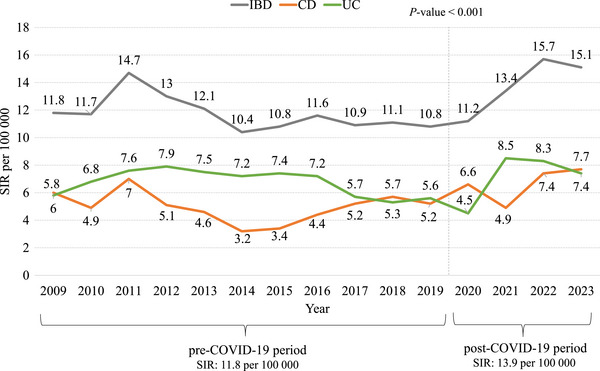
Annual standardized incidence rates (SIRs) per 100 000 persons aged 0–14 years by type of IBD, Apulia region, Italy, 2009–2023. IBD, inflammatory bowel disease; CD, Crohn's disease; COVID‐19, coronavirus disease 2019; UC, ulcerative colitis.

In the period 2009–2023, the average age‐standardized incidence was 12.7 (95% CI: 11.9–13.5) per 100 000 persons/years. Annual SIR increased from 2020 onward (2020–2023: 13.9 per 100 000 vs. 2009–2019: 11.8 per 100 000; *P*‐value < 0.001). Average SIR was 13.3 per 100 000 (95% CI: 12.2–14.4) in males and 12.0 per 100 000 (95% CI: 10.9–13.1) in females. The highest SIR was observed in the 10–14 years age group (7.3 per 100 000; 95% CI: 6.7–7.8) (Tables S2 and S3). The annual SIR of CD in the period 2009–2023 was 5.6 per 100 000 (95% CI: 5.1–6.1), ranging from 3.2 per 100 000 in 2014 to 7.7 per 100 000 in 2023, with a significant increase observed from 2020 onward (*P*‐value < 0.0001). SIR of CD was 6.3 per 100 000 (95% CI: 5.6–7.1) in males and 4.8 per 100 000 (95% CI: 4.1–5.5) in females. The highest SIR was observed in the 10–14 years age group (3.2 per 100 000; 95% CI: 2.9–3.6). The average SIR of UC from 2009 to 2023 was 7.1 per 100 000 (95% CI: 6.5–7.7), ranging from 4.5 per 100 000 in 2020 to 8.5 per 100 000 in 2021 (*P*‐value < 0.0001; Figure 2, Tables S2 and S3). In males, the rate was 6.9 per 100 000 (95% CI: 6.2–7.8) and 7.2 per 100 000 (95% CI: 6.4–8.0) in females. The highest SIR was observed in the 10–14 years age group (4.0 per 100 000, 95% CI: 3.6–4.5; Table S3).

To assess vaccination coverage, 259 patients aged 0–14 years on December 31, 2024, were selected. Vaccination coverage rates for hexavalent full vaccination (DTaP‐IPV‐HBV‐Hib) cycle was 100% in both the CD and UC groups. PCV coverage was 99.2% overall (99.1% CD, 99.3% UC), rotavirus was 82.5% overall (81.8% CD, 82.9% UC), and MenB was 79.9% overall (78.9% CD, 80.7% UC). MenC coverage was 83.9% overall (89.2% CD, 79.7% UC) in infants, 89.7% in infants (83.3% CD, 94.1% UC), and 55.9% in adolescents (51.8% CD, 59.6% UC). MMRV or MMR+V coverage was 87.6% overall (86.5% CD, 88.4% UC), and HAV was 87.6% overall (87.7% CD, 87.6% UC). HPV vaccination coverage was 66.3% in females overall (62.9% CD, 68.8% UC) and 52.1% in males overall (50.0% CD, 54.3% UC). The overall dTap‐IPV booster coverage was 30.2% (31.3% CD, 29.4% UC). The seasonal influenza vaccination coverage ranged from 20.8% in 2020/2021 to 18.9% in 2023/2024. COVID‐19 vaccination coverage (two‐dose schedule) was 47.2% (46.8% CD, 47.4% UC; Table 2). Children with complex chronic conditions were significantly less likely to receive the first booster of DTaP‐IPV/dTap‐IPV (*P* = 0.003) or MMR/MMR+V (*P* = 0.028; Table 3).

**TABLE 2:** Vaccination coverage for childhood and adolescent vaccines in currently pediatric IBD Patients (0–14 years) by diagnosis, Apulia Region, Italy, 2009–2023

	CD			UC		
Vaccines	N. eligible	N. vaccinated	VC (%)	N. eligible	N. vaccinated	VC (%)
DTaP‐IPV‐HBV‐Hib	114	114	100.0	145	145	100.0
PCV	114	113	99.1	145	144	99.3
Rotavirus	22	18	81.8	35	29	82.9
MenB	114	90	78.9	145	117	80.7
MenC	102	91	89.2	128	102	79.7
MenACWY^a^	12	10	83.3	17	16	94.1
MenACWY^b^	83	43	51.8	94	56	59.6
HAV	114	100	87.7	145	127	87.6
DTaP‐IPV/dTap‐IPV^c^	112	100	89.3	138	130	94.2
MMRV or MMR+V	111	96	86.5	138	122	88.4
HPV female	35	22	62.9	48	33	68.8
HPV male	48	24	50.0	46	25	54.3
dTap‐IPV^d^	48	15	31.3	68	20	29.4
2020–2021 flu season	114	21	18.4	145	33	22.8
2021–2022 flu season	114	22	19.3	145	34	23.4
2022–2023 flu season	114	21	18.4	145	29	20.0
2023–2024 flu season	114	20	17.5	145	29	20.0
COVID‐19^e^	111	52	46.8	137	65	47.4

*Note*: 259 patients aged 0–14 years on December 31, 2024, were selected. Ten subjects were excluded from the analysis because their vaccination status could not be retrieved, as they were not listed in the regional vaccination registry because they had moved to another region.

^a^ 15 months of age.

^b^ Aged 11–12 years.

^c^ Aged 5–6 years.

^d^ Aged 13–14 years.

^e^ Aged ≥5 years, 2021–2023.

Abbreviations: COVID‐19, coronavirus disease 2019; DTaP/dTap, diphtheria, tetanus, and acellular pertussis; flu, influenza; HAV, hepatitis viruses A; HBV, hepatitis viruses B; Hib, *Haemophilus influenzae* type b; HPV, human papillomavirus; IPV, inactivated polio vaccines; MMR, measles, mumps, and rubella; Men, meningococcal; PCV, pneumococcal conjugate vaccines; V, varicella; VC, vaccination coverage.

**TABLE 3:** Univariate/multivariate analysis assessing association between vaccination status and complex chronic conditions in currently pediatric inflammatory bowel disease (IBD) patients (0–14 years), Apulia Region, Italy, 2009–2023

	Univariate analysis			Multivariate analysis		
Vaccines	OR	95% CI	*P*‐value	OR	95% CI	*P*‐value
PCV	−	−	0.504	−	−	−
Rotavirus	0.82	0.12–9.38	0.822	−	−	−
MenB	1.53	0.62–4.34	0.327	−	−	−
MenC	0.88	0.34–2.59	0.789	−	−	−
MenACWY	1.03	0.48–2.27	0.932	−	−	−
HAV	0.51	0.21–1.36	0.118	−	−	−
DTaP‐IPV/dTap‐IPV^a^	0.23	0.08–0.69	0.001	0.22	0.08–0.60	0.003
MMRV or MMR+V	0.40	0.16–1.04	0.031	0.38	0.15–0.89	0.028
HPV	0.97	0.41–2.32	0.931	−	−	−
dTap‐IPV^b^	0.73	0.19–2.39	0.580	−	−	−
At least one flu season	1.20	0.59–2.43	0.572	−	−	−
COVID‐19^c^	0.97	0.48–1.95	0.940	−	−	−

^a^ Aged 5–6 years.

^b^ Aged 13–14 years.

^c^ Aged ≥5 years, 2021–2023.

Abbreviations: CI, confidence interval; COVID‐19, coronavirus disease 2019; DTaP/dTap, diphtheria, tetanus, and acellular pertussis; flu, influenza; HAV, hepatitis viruses A; HPV, human papillomavirus; IPV, inactivated polio vaccines; Men, meningococcal; MMR, measles, mumps, and rubella; OR, odds ratio; PCV, pneumococcal conjugate vaccines; V, varicella.

## DISCUSSION

Using administrative database analyses, the present study examined the epidemiological landscape of pediatric IBD in the region of Apulia, a large geographical area in southern Italy, over a 14‐year period (2009–2023).

The reported SIRs were consistent with those reported in Western Europe during the first two decades of the 21^st^ century, ranging from 5.4 to 17.4 per 100 000 person/years for IBD, 2.1–15.3 per 100 000 for CD, and 1.5–18.4 per 100 000 person/years for UC with differences possibly explained by different study designs, data collection methods, and data sources.^3^ Similarly, the estimated SIRs in the Apulian region were in line with those observed in recent Italian population‐based studies.^12^, ^36^ In particular, in a study conducted between 2015 and 2018, Crocetti et al.^12^ reported an SIR for CD of just under 2 per 100 000 in children aged 0–4 years, rising to just over 2 in 5–9‐year‐olds, and reaching around 4 in the 10–14 years age group. The incidence of UC showed a similar upward trend, with rates reaching just over 5 per 100 000 in children aged 10–14 years. Piscaglia et al.^36^ reported an SIR of 9.1 for UC and 6.1 for CD in patients under 20 years of age in 2010–2014. In addition, a significant increase in incidence was observed since the beginning of the COVID‐19 pandemic, consistent with data from a recent systematic review in which 84% of reported studies showed a similar trend.^3^ This rise in pediatric IBD cases is likely to have been driven by several potential environmental factors acting on a genetic predisposition. The increasing westernization of diets may have exposed a widespread genetic vulnerability that predisposes individuals to different etiologies of the disease.^7^ Moreover, it has been hypothesized that severe acute respiratory syndrome coronavirus 2 (SARS‑CoV‑2) infection may play a role in the development of IBD by altering the gut microbiota and promoting inflammation in genetically susceptible individuals.^37^ Another contributing factor may have been the underdiagnosis of cases during the lockdown period due to restricted access to health services, leading to a backlog of diagnoses in subsequent years.^4^

A slight, not statistically significant, male predominance in the incidence of pediatric IBD was observed in this study. This trend is supported by most reports of CD, where the disease is more common in male children, whereas the incidence of UC tends to be more balanced between the sexes. Research suggests that males are more likely to be diagnosed with IBD than females, with a reported male‐to‐female ratio of 2.2:1 for CD.^4^, ^38^ Males have a more extensive and severe disease phenotype and may carry genetic markers that increase susceptibility to IBD, particularly CD.^39^ Population‐level data from 16 Western countries have suggested that, until puberty, females have a lower risk of developing CD than males, possibly due to sex hormone‐dependent mechanisms.^38^, ^40^ Additionally, sex‐related differences in the gut microbiome have been increasingly recognized as contributing factors in various diseases, including IBD.^38^

In the present study, the incidence of IBD was higher in older children (pre‐adolescents) than in younger children. Similarly, to other authors,^41^, ^42^ Kern et al.^43^ reported an increasing trend from early childhood to adolescence, with IBD incidence rising from 2.3 per 100 000 in the 0–4 age group to 14.3 per 100 000 in the 10–14 age group.^43^ Several factors have been implicated in this rising trend, including dietary shifts towards ultra‐processed foods, infections, use of non‐steroidal anti‐inflammatory drugs, exposure to antibiotics, and smoking.^44^ The increasing burden of IBD among adolescents underscores the need for urgent attention to prevention strategies and optimized healthcare management. Conversely, although IBD is less frequently reported in infants, early‐onset cases can lead to significant long‐term health challenges, highlighting the importance of early diagnosis, intervention, and specialized care.^41^, ^42^, ^43^, ^44^

Several comorbid conditions have been associated with pediatric IBD, including cardiovascular diseases, neuropsychological disorders, and metabolic syndromes. Research has highlighted a wide range of comorbidities, from immune‐mediated diseases to serious infections, highlighting the multifaceted nature of IBD. The strongest associations were with other immune‐mediated inflammatory diseases, probably because of the shared pathogenic pathways. Conditions such as rheumatoid arthritis, systemic lupus erythematosus, type 1 diabetes, psoriasis, asthma, celiac disease, and hypothyroidism are more commonly reported in children with IBD.^45^ The prevalence reported among IBD incident cases in the Apulia region was in line with Richard et al.,^46^ who estimated that 26.9% of pediatric IBD patients have comorbid conditions. In particular, CD is often associated with chronic gastritis, irritable bowel syndrome, and allergic diseases such as atopic dermatitis and allergic rhinitis, suggesting a possible link between autoimmune and allergic responses. Consistent with the Apulian data, anemia is one of the most common extraintestinal manifestations of IBD, resulting from chronic inflammation, iron sequestration, and malabsorption of essential nutrients such as iron, vitamin B12, and folate.^47^ In addition to abdominal pain and diarrhea, children with UC often suffer from dehydration and nutritional deficiencies due to frequent and sometimes bloody diarrhea.^48^ Given these challenges, early diagnosis, multidisciplinary management, and nutritional interventions are critical to improving the long‐term health outcomes of pediatric IBD patients.

In contrast to those reported in other contexts,^14^, ^49^ there was generally good compliance with the vaccinations recommended by the Apulian vaccination schedule, with suboptimal uptake for influenza, the second dose of dTap‐IPV, MenACWY, and HPV. For example, the two‐dose MMR vaccination coverage in the general population of Apulia ranged from 82.6% to 91.5% for the 2010–2016 birth cohorts, which is consistent with the 87.6% of children with IBD evaluated in this study. Moreover, a comparison with official HPV vaccination coverage data from the Puglia region for the most recent cohort (born in 2011) showed that females with IBD had higher coverage than females in the general population (66.3% vs. 53.8%). Increased awareness of the increased risk of HPV‐related complications may explain the higher HPV vaccination coverage observed in female IBD patients, although it is noteworthy that males with IBD were more likely to be vaccinated than their counterparts in the general population (52.1% vs. 48.5%). Furthermore, compared with the general Italian population,^50^ children and adolescents with IBD showed a slightly higher uptake of the SARS‐CoV‐2 vaccine (47.2% vs. approximately 40% among people under 20 and 34% among children aged 5–11, as of November 2023), suggesting that targeted recommendations or closer clinical follow‐up could improve. These findings are encouraging, as they suggest that chronic conditions such as IBD do not appear to negatively impact adherence to key childhood vaccinations and may reflect effective communication and proactive vaccination strategies within the care pathways for pediatric IBD patients in the Apulia region of Italy. Nevertheless, overall vaccination coverage for HPV remains suboptimal, particularly among male adolescents, primarily due to limited parental awareness.^51^, ^52^ Tailored and inclusive strategies are essential to ensure equitable protection across genders and reduce the overall burden of HPV‐related morbidity. Likewise, the consistently low coverage of seasonal influenza vaccination over the four seasons represents an area in need of improvement, which could be achieved through enhanced awareness campaigns and better integration of influenza vaccination into routine care pathways for children with IBD.

The findings in relation to complex chronic conditions, particularly the lower likelihood of receiving the first booster of DTaP‐IPV/dTap‐IPV and MMR/MMR+V. This may indicate barriers to vaccine access, particularly for children with more complex health needs, such as provider concerns, missed appointments, or underlying issues related to the management of these chronic conditions. As suggested by Fleurier et al.,^53^ simply informing IBD patients, their parents, and general practitioners about the vaccination schedule by post may be a simple, cost‐effective, and reproducible strategy to increase vaccination coverage, especially in children with IBD who benefit more from immunization. In addition, improving the immunization information system to include information on patients' chronic conditions may facilitate timely reminders and ultimately enhance adherence rates.

This study has several limitations. Incidence estimates were based on health registries, which may be subject to misclassification errors, underreporting, or incomplete data entry. The use of hospital data might have underestimated the true incidence by excluding milder cases. Although integrating the UFER registry with HDR increased sensitivity, it is important to acknowledge that not all eligible patients apply for exemption, which could lead to under‐ascertainment. Furthermore, registry completeness can differ between regions and over time, and the exemption date may not correspond to the actual clinical diagnosis.^54^ The regional perspective of the study could result in small sample sizes in certain subgroups, which could lead to wide confidence intervals when calculating IRRs. Owing to the unavailability of outpatient data, the assessment of comorbidities was limited to patients previously hospitalized in the Puglia region. Moreover, the available sources were unable to integrate information useful for assessing disease severity, specific treatment regimens, or individual risk factors that may have influenced vaccination decisions. This represents a significant limitation, as immunosuppressive therapies directly determine vaccination eligibility and timing according to the current guidelines. It is likely that vaccination coverage differs substantially between patients receiving immunosuppressive therapy and those with mild disease. However, therapeutic stratification was not observed in the present study. The timing and type of therapy can affect the suitability of certain vaccines (e.g., live attenuated vaccines) as well as when they should be administered, implying complex coordination between specialists and vaccination services. Future studies should aim to integrate clinical data from hospital or specialist patient records to more effectively elucidate the relationship between therapeutic management and vaccine uptake.

In conclusion, this study provides updated data on the incidence of pediatric IBD in the Apulia region of Italy, confirming an increasing trend over the last decade, which is consistent with global epidemiological patterns, and highlights the growing burden of IBD in children. Although vaccination coverage among pediatric IBD patients in Apulia appears comfortable, room for improvement remains. Future research is needed to further investigate the barriers in this high‐risk population, assess longitudinal vaccination trends, and evaluate the effectiveness of targeted interventions to improve vaccine uptake. A multidisciplinary approach involving pediatricians, gastroenterologists, and public health specialists is crucial in optimizing preventive care strategies for children with IBD.

## CONFLICT OF INTEREST

The authors declare no conflict of interest.

## Supporting information



Supporting Information
