# Supplementary material for: A 15‐year population‐based study on incidence and vaccination coverage in pediatric inflammatory bowel disease in Italy
Source: Pediatr Investig. 2025 Sep 18;9(4):383–93. doi: 10.1002/ped4.70023 (PMC12715895; doi:10.1002/ped4.70023)
Supplement: Supplementary file 1 — Supporting Information [file PED4-9-383-s001.pdf]

Supplementary Material for

A 15-Year population-based study on incidence and vaccination coverage in pediatric inflammatory bowel disease in Italy

Francesca Fortunato, Angelo Campanozzi, Michele Di Toma, Alessandra Marinari, Domenico Martinelli

Table S1. Vaccination schedules by birth cohort included in the vaccination coverage estimates, according to the evolution of the regional immunisation plan in Apulia Region, Italy, 2010-2023

**Vaccination**	**Schedule (doses)**	**Target population (age)**	**Year of introduction**	**Birth cohorts**													
				**2010**	**2011**	**2012**	**2013**	**2014**	**2015**	**2016**	**2017**	**2018**	**2019**	**2020**	**2021**	**2022**	**2023**
DTaP-IPV-HBV-Hib	2+1	Infants (0–12 months)	2001	X	X	X	X	X	X	X	X	X	X	X	X	X	X
PCV	3	Infants (0–12 months)	2006	X	X	X	X	X	X	X	X	X	X	X	X	X	X
Rotavirus	2 or 3	Infants (0–8 months)	2011 for infants with chronic conditions; 2018 for all infants		X	X	X	X	X	X	X	X	X	X	X	X	X
MenB	3+1<br>2+1 since 2021	Infants (0–15 months)	2014 for all infants; 2017 for all infants, with catch-up for birth cohorts 2014–2016					X	X	X	X	X	X	X	X	X	X
MenB	1	Adolescents (11–12 years)	2018	X	X	X	X										
MenC	1	Infants (15 months)	2006	X	X	X	X	X	X	X							
MenACWY	1	Infants (15 months)	2017								X	X	X	X	X	X	X
MenACWY	1	Adolescents (11–12 years)	2011	X	X	X	X										
HAV	2	Children (13–24 months)	1998	X	X	X	X	X	X	X	X	X	X	X	X	X	X
Dtap-IPV/dTap-IPV	1	Children (5–6 years)	2012	X	X	X	X	X	X	X	X	X	X				
MMRV or MMR+V	1+1	Children (13 months)<br>Children (5–6 years)	MMR before 2009<br>V (one shot): 2006 – V (2 shots): 2009	X	X	X	X	X	X	X	X	X	X	X	X	X	X
HPV	2	Adolescents (11–12 years)	2008 in female; 2014 in male	X	X	X	X										
dTapIPV	1	Adolescents (13–14 years)	2012	X	X												
COVID-19	2	Children (≥5 years)	2021	X	X	X	X	X	X	X	X	X					

DTaP-IPV-HBV-Hib: hexavalent diphtheria, tetanus, acellular pertussis, inactivated poliovirus, hepatitis B, *Haemophilus influenzae* b conjugate vaccine
PCV: pneumococcal conjugate vaccine
MenB: Meningococcal B
MenC: Meningococcal C
MenACWY: Meningococcal ACWY
HAV: hepatitis A virus
DTap-IPV/dTap-IPV: diphtheria, tetanus, acellular pertussis, inactivated poliovirus
MMRV: measles, mumps, rubella, varicella
HPV: human papilloma virus
PPSV23: pneumococcal plysaccharide vaccine
Reference: 27-33

Table S2. Annual age-standardized incidence rates (SIR) per 100,000 persons aged 0-14 years, by type of IBD, sex, year, and age group. Apulia region, Italy, 2009–2023

Varibles	CD						UC						IBD					
	All		Male		Female		All		Male		Female		All		Male		Female	
	SIR	95% CI	SIR	95% CI	SIR	95% CI	SIR	95% CI	SIR	95% CI	SIR	95% CI	SIR	95% CI	SIR	95% CI	SIR	95% CI
**Year**																		
**2009**	6.0	4.1, 7.9	6.3	3.5, 9.0	5.6	3.0, 8.3	5.8	3.9, 7.7	6.9	4.0, 9.8	4.6	2.2, 7.1	11.8	9.1, 14.5	13.2	9.2, 17.2	10.3	6.7, 13.9
**2010**	4.9	3.1, 6.6	5.4	2.8, 7.9	4.4	2.0, 6.7	6.8	4.7, 8.9	7.2	4.3, 10.2	6.3	3.5, 9.2	11.7	9.0, 14.4	12.6	8.7, 16.5	10.7	7.0, 14.4
**2011**	7.0	4.9, 9.1	8.0	4.8, 11.1	6.0	3.2, 8.8	7.6	5.5, 9.8	7.0	4.1, 9.9	8.4	5.1, 11.6	14.7	11.6, 17.7	15	10.7, 19.2	14.4	10.1, 18.7
**2012**	5.1	3.3, 6.9	6.7	3.8, 9.5	3.4	1.3, 5.5	7.9	5.7, 10.2	8.7	5.4, 12.0	7.1	4.1, 10.2	13.0	10.2, 15.9	15.4	11.0, 19.7	10.6	6.8, 14.3
**2013**	4.6	2.9, 6.4	3.5	1.4, 5.6	5.8	3.0, 8.6	7.5	5.3, 9.7	7.7	4.6, 10.8	7.2	4.1, 10.3	12.1	9.3, 14.9	11.3	7.5, 15.0	13.0	8.9, 17.2
**2014**	3.2	1.7, 4.6	3.2	1.2, 5.3	3.1	1.1, 5.1	7.2	5.1, 9.4	8.1	4.9, 11.3	6.2	3.4, 9.1	10.4	7.8, 13.0	11.4	7.6, 15.1	9.4	5.8, 12.9
**2015**	3.4	1.9, 4.9	3.7	1.5, 5.8	3.2	1.1, 5.2	7.4	5.2, 9.6	6.6	3.7, 9.5	8.2	4.8, 11.6	10.8	8.1, 13.5	10.3	6.7, 13.9	11.3	7.4, 15.3
**2016**	4.4	2.7, 6.1	4.2	1.8, 6.5	4.7	2.1, 7.2	7.2	5.0, 9.4	7.6	4.4, 10.8	6.7	3.7, 9.7	11.6	8.8, 14.4	11.8	7.8, 15.7	11.4	7.4, 15.3
**2017**	5.2	3.3, 7.1	5.6	2.9, 8.4	4.7	2.2, 7.3	5.7	3.7, 7.7	4.9	2.3, 7.5	6.6	3.5, 9.6	10.9	8.2, 13.6	10.5	6.7, 14.3	11.3	7.3, 15.3
**2018**	5.7	3.7, 7.7	8.3	4.9, 11.6	3.0	0.9, 5.1	5.3	3.4, 7.3	5.4	2.7, 8.1	5.3	2.5, 8.0	11.1	8.3, 13.9	13.7	9.3, 18.0	8.3	4.8, 11.8
**2019**	5.2	3.3, 7.1	5.4	2.7, 8.1	5.0	2.3, 7.8	5.6	3.6, 7.7	4.8	2.2, 7.4	6.5	3.4, 9.6	10.8	8.0, 13.6	10.2	6.4, 14.0	11.5	7.4, 15.7
**2020**	6.6	4.4, 8.8	7.7	4.4, 11.0	5.5	2.6, 8.4	4.5	2.7, 6.4	4.3	1.9, 6.8	4.8	2.1, 7.5	11.2	8.3, 14.0	12.0	7.9, 16.1	10.3	6.3, 14.2
**2021**	4.9	3.0, 6.8	5.5	2.7, 8.3	4.3	1.7, 6.8	8.5	6.0, 11.0	8.7	5.1, 12.3	8.3	4.7, 11.9	13.4	10.3, 16.6	14.2	9.7, 18.7	12.6	8.2, 16.9
**2022**	7.4	5.0, 9.7	9.3	5.7, 13.0	5.3	2.4, 8.1	8.3	5.8, 10.8	6.8	3.6, 9.9	9.9	6.0, 13.9	15.7	12.2, 19.1	16.1	11.3, 20.9	15.2	10.4, 20.1
**2023**	7.7	5.3, 10.1	9.3	5.7, 13.0	5.9	2.9, 9.0	7.4	5.0, 9.7	6.5	3.4, 9.6	8.3	4.6, 11.9	15.1	11.7, 18.4	15.8	11.0, 20.6	14.2	9.5, 18.9
**Age group (years)**																		
**0-4**	0.8	0.6, 1.0	0.8	0.5, 1.1	0.7	0.4, 1.0	1.0	0.8, 1.2	1.0	0.7, 1.4	1.0	0.7, 1.3	1.8	1.5, 2.1	1.9	1.5, 2.3	1.7	1.3, 2.1
**5-9**	1.4	1.1, 1.6	1.5	1.1,1.9	1.3	0.9, 1.6	1.8	1.5, 2.1	1.7	1.3, 2.1	1.9	1.5, 2.4	3.2	2.8, 3.6	3.2	2.7, 3.8	3.2	2.6, 3.7
**10-14**	3.2	2.9, 3.6	3.8	3.2, 4.3	2.7	2.2, 3.2	4.0	3.6, 4.5	4.0	3.5, 4.6	4.0	3.4, 4.6	7.3	6.7, 7.8	7.8	7.0, 8.6	6.7	6.0, 7.5
**Total**	5.6	5.1, 6.1	6.3	5.6, 7.1	4.8	4.1, 5.5	7.1	6.5, 7.7	6.9	6.2, 7.8	7.2	6.4, 8.0	12.7	11.9, 13.5	13.3	12.2, 14.4	12.0	10.9, 13.1

IBD, inflammatory bowel disease; CD, Crohn’s disease; UC, ulcerative colitis; CI, confidence intervals.

Table S3. Incidence rate ratios, by type of IBD, pre-COVID-19 period (2009-2019) and the post-COVID-19 period (2020-2023), sex and age group. Apulia region, Italy, 2009–2023

	IBD				CD				UC			
**Variables**	**IRR**	**95% CI**	***z***	***P*-value**	**IRR**	**95%CI**	***z***	***P*-value**	**IRR**	**95% CI**	***z***	***P*-value**
Period [Post-COVID-19 (2020-2023) vs Pre-COVID-19 (2009-2019)]	3.7	1.7, 8.1	3.3	<0.001	4.3	1.3, 13.8	4.1	<0.001	3.4	1.2, 9.7	2.3	<0.001
Sex (Female vs Male)	0.9	0.4, 2.1	−0.1	0.907	0.8	0.2, 2.6	−0.6	0.722	1.1	0.4, 3.1	0.2	0.853
Age (10–14 yrs vs 0–4 yrs)	3.3	0.6, 17.0	1.4	0.148	3.3	0.3, 38.1	0.9	0.341	3.3	0.4, 29.4	0.9	0.287

IBD, inflammatory bowel disease; CD, Crohn's disease; UC, ulcerative colitis; CI, confidence intervals; IRR, incidence rate ratios.
